# Analysis of Quality of Life and Nutritional Status in Elderly Patients with Dysphagia in Order to Prevent Hospital Admissions in a COVID-19 Pandemic

**DOI:** 10.3390/life11010022

**Published:** 2020-12-31

**Authors:** Virginia E. Fernández-Ruiz, Rocío Paredes-Ibáñez, David Armero-Barranco, Juan Francisco Sánchez-Romera, Mercedes Ferrer

**Affiliations:** 1 Department of Endocrinology and Nutrition, Virgen de la Arrixaca University Clinic Hospital, 30120 Murcia, Spain; virginiaesperanza.fernandez@um.es (V.E.F.-R.); mercedesferrer@um.es (M.F.); 2 Faculty of Nursing, Calle Campus Universitario, University of Murcia, 11, 30100 Murcia, Spain; darmero@um.es; 3 Community and Family Nursing Specialist, Calle Campus Universitario, University of Murcia, 11, 30100 Murcia, Spain; 4 Department of Human Anatomy and Psychobiology, Calle Campus Universitario, University of Murcia, 11, 30100 Murcia, Spain; juanfrancisco.sanchez@um.es; 5 Endocrinology and Nutrition Department, Calle Campus Universitario, University of Murcia, 11, 30100 Murcia, Spain

**Keywords:** oropharyngeal dysphagia, elderly people, quality of life, thickener, malnutrition

## Abstract

(1) Background: Oropharyngeal dysphagia (OD) is currently recognized as one of the geriatric syndromes due to its high frequency in older people and its associated complications, which have a direct impact on quality of life. The main objective is to determine the effectiveness of telehealth consultation for the re-evaluation of nutritional status and quality of life assessment in older people diagnosed with OD associated with active use of thickeners to prevent hospital admissions in a COVID-19 pandemic. (2) Methods: an observational, descriptive, and longitudinal study that included a sample of 33 subjects with age equal or superior to 65 years diagnosed with OD with conserved cognitive capacity. The nutritional status was evaluated through the Mini-Nutritional Assessment (MNA) questionnaire and biochemical parameters and, the quality of life was determined through the Swallowing Quality of Life (SWAL-QOL) questionnaire. (3) Results: Thirty-three older patients with OD were recruited (54.5% women), with a mean age of 83.5 ± 7.6 years. The main cause of OD in the study population was neurodegenerative disease (51.5%), followed by cerebrovascular disease (33.3%), and other causes (15.2%). Sixty point six percent of patients were found to be at risk of malnutrition. The MNA score was significantly correlated to albumin (r: 0.600, *p* < 0.001) and total proteins (r: 0.435, *p* = 0.015), but not to total cholesterol (r: −0.116, *p* = 0.534) or lymphocytes (r: −0.056, *p* = 0.758). The mean total score of the SWAL-QOL was 75.1 ± 16.4 points. (4) Conclusions: the quality of life of the subjects related to the use of a thickener is good. Although the body mass index (BMI) and average biochemical, nutritional parameters of the subjects are within the range of normality, the MNA has detected a high percentage of subjects with the risk of malnutrition, which suggests the need for continuous re-evaluation in these patients, demonstrating the viability of the telematic route in this research.

## 1. Introduction

Oropharyngeal dysphagia (OD), or difficulty swallowing, is a common clinical symptom among older people [1,2]. Although aging in itself is not a cause of clinically evident OD, there are age-related changes that can affect all phases of swallowing [1,2,3,4]. Indicators of OD in healthy older people include the lack of teeth, esophageal weakness, and decreased cognitive function [3]. Accordingly, OD has been considered an indicator of frailty [3] and dependence [4] and is currently recognized as a geriatric syndrome [5,6]. Currently, the prevalence of OD in the world population is 16–23% and increases to 30% in older people [7,8,9]; it increases even further in the presence of dementia [4,6], reaching a prevalence of up to 85.9% in Spain [10]. In Europe, the prevalence of OD in the over-65s is 40%, and in institutionalized elderly people, it is more than 60% [11].

Although the prevalence in elderly patients is high and has a great impact on functional quality and morbimortality, it is one of the least known and most underestimated geriatric syndromes [1,3]. Different authors [4,6,12] underscore that OD is underdiagnosed due to the tendency of older patients to underestimate their swallowing problems, and caregivers are thus often unable to detect them. These factors contribute to the appearance of complications, reduced life expectancy, and increased healthcare costs [1,13,14].

Complications of OD include malnutrition [3,15], dehydration [3,15], functional impairment [15,16], and aspiration pneumonia [3,15,17]—the latter being a major cause of morbidity and mortality due to infection in older people [1,3,15]. Furthermore, two of the most prominent consequences of OD are its impact on the quality of life of older people [3,16,18] and the increased burden it places on the caregiver [1,19].

Multidisciplinary rehabilitation teams are needed to provide complementary expertise in the treatment of swallowing disorders (endocrinologists, nurses, phoniatrists, and speech-language therapists, endocrinologists, gastroenterologists, physiatrists), who contribute to swallowing assessment (clinical and instrumental) and treatment interventions [20,21]. On the other hand, dieticians and nutritionists are responsible for making recommendations for adequate caloric and nutritional intake when modification of diet texture or non-oral feeding is necessary [22]. Occupational therapists will also contribute to the improvement in eating and food-related behaviors [21]. This subject becomes all the more relevant at the present time in the context of the COVID-19 pandemic, where in anticipation of a second wave of the disease, it is a priority to keep the assistance safe, through telepathic consultation (thelesalud), to avoid hospital admissions of elderly dysphagic patients due to respiratory infections caused by aspiration or other complications of OD, such as malnutrition and dehydration. The main objective is to determine the effectiveness of telehealth consultation for the re-evaluation of nutritional status and quality of life assessment in older people diagnosed with OD associated with the active use of thickeners to prevent hospital admissions in the COVID-19 pandemic.

## 2. Materials and Methods

### 2.1. Ethical Considerations

The study was approved by the Ethics Committee of the Virgen de la Arrixaca University Clinic Hospital (VAUCH) (Murcia, Spain) (Ref.: 2020-6-14-HCUVA). The research was carried out under the ethical principles of the Declaration of Helsinki, ensuring complete anonymity and confidentiality of the data and information about the analyzed patients, acting in accordance with Spanish Law 3/2018 of 5 December, on the Protection of Personal Data.

### 2.2. Study Design and Participants

An observational, descriptive, longitudinal study was carried out. The study population was composed of patients over 65 years of age diagnosed with OD from Healthcare Area I (Murcia, Spain). The patients were followed-up on by the Nutrition Unit of Virgen de la Arrixaca University Clinic Hospital (VAUCH) Endocrinology and Nutrition by those patients who had a thickener prescription, approved through the NED clinical route. In addition, in the case of patients with some type of dependency implying the need for a caregiver, the latter was included in the study to establish the degree of burden—with the signing of the informed consent being requested from both (patient and caregiver). Healthcare Area I (Murcia Oeste) covers a population of 266,460 individuals, according to the 2019 census. Data from the regional statistics authority (Centro Regional de Estadística de Murcia (CREM)) show this figure to correspond to 17.8% of the total inhabitants of the region of Murcia. Of the mentioned individuals, 41,150 were over 65 years old (15.44%), and women represented 56.82%.

Patients with a prescription for thickener of less than 6 months were excluded from the study, in the same way as those who, at the time of the study, did not use the thickener, patients with cognitive disorders making it impossible to answer the questionnaires or those who, of their own free will, declined to participate in the study and, therefore, did not sign the informed consent form.

**Figure 1:**
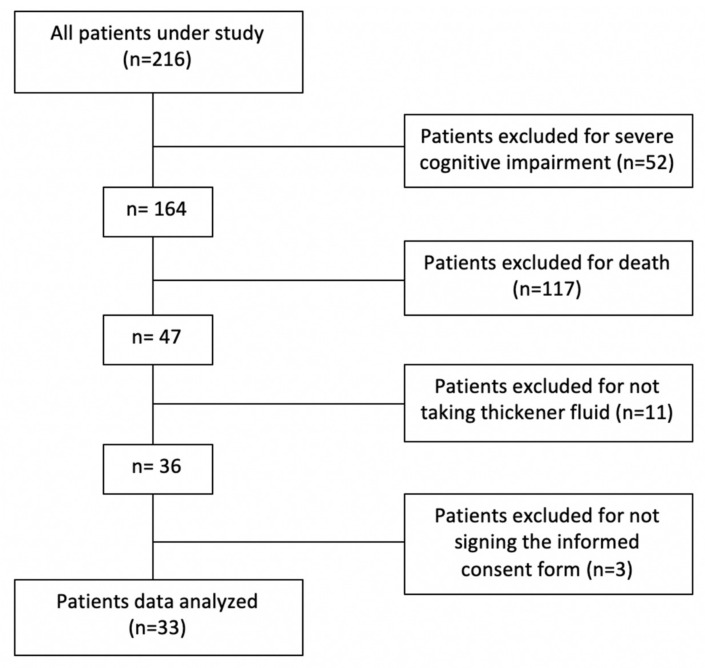
Study flow chart.

Of the total population under study (216 subjects informed from the Central Service), only 33 met the inclusion criteria (Figure 1). The prevalence of patients with active prescription of thickener and OD was 2.14% in the study Healthcare Area. All the patients have been diagnosed with OD in a previous visit to the vacuum nutrition unit in person by March 2020. They were diagnosed with OD through the MECV-V, and the texture of the thickener to be used was indicated.

The nutrition unit (endocrine, nutritionist, nurse, and psychologist), on the day of the diagnosis, carried out a training session with the patient and his/her main caregiver. We explained to them the impact of the OD; written recommendations were made and given for the use of the thickener, behavioral and nutritional recommendations for the patient with OD, and alarm signs that compromised safety and indicated the progression of OD (cough, throat clearing, wet voice, dysphonia). In addition, simple exercises created by the rehabilitation service were provided to strengthen the swallowing muscles. On the same day, a consultation was made with the rehabilitation service (rehabilitation doctor, speech therapist, and physiotherapist) so that the patient could receive complete rehabilitation treatment.

The study was conducted during the months of August and September 2020 by the interdisciplinary team of the Nutrition Unit of VAUCH, composed of endocrinologists, nurses, nutritionists, and psychologists. Bearing in mind the characteristics, the state of vulnerability, and, therefore, the risk of the population under analysis, the decision was made to carry out the study by video call to avoid physical contact. During the first week of August, all patients were informed verbally (by phone call) and in writing (by e-mail) of the study, leaving them a week to sign and return the informed consent document (via e-mail).

### 2.3. Instruments and Measurement Procedure

The study took place within the context of the COVID-19 pandemic, with the suspension of face-to-face consultations for patients at risk. The Nutrition Unit of the VAUCH proposed a non-physical presence and objective re-evaluation of the nutritional status and quality of life of all elderly patients with OD, together with an evaluation of the extra burden their main caregivers were experiencing.

Other variables collected were age, sex, type of diet, use of supplements, dentition, edemas, muscle mass loss, arterial hypertension (AHT), diabetes mellitus (DM), aspiration pneumonia, primary diagnosis (stroke, neurodegenerative disease, etc.), type of caregivers, and sex of caregivers.

Nutritional status was assessed using the Mini-Nutritional Assessment (MNA) questionnaire—a noninvasive, validated screening tool for the early detection of malnutrition or the risk of malnutrition in the elderly [23]. The MNA is composed of 18 variables that include, among others, anthropometric parameters (weight loss, body mass index (BMI), brachial circumference (BC), and calf circumference (CC)), assessment of dietary intake (qualitative and quantitative food intake), a global assessment of lifestyle and functionality (medication, mobility, presence of dementia, depression, acute stress) and self-assessment. The assessment of nutritional status was carried out using a scale where a score of 24–30 points indicated good nutritional status, 17–23.5 points indicated a risk of malnutrition, and a score of <17 points indicated malnutrition. To complete the assessment of nutritional status, we evaluated a number of analytical parameters (typical general biochemical analysis in nutrition practice), as well as other variables considered of interest in relation to nutritional status (oral nutritional supplementation (ONS), dental status, objective loss of muscle mass and presence of edema).

The Spanish version of the Swallowing Quality of Life questionnaire (SWAL-QOL) was used for the evaluation of the quality of life related to OD. The SWAL-QOL consists of 44 questions subdivided into 11 domains: general caregiver burden, duration of intake, appetite, frequency of symptoms, food selection, communication, fear of eating, mental health, social functioning, fatigue, and sleep. The scoring system is based on an index from 0–100, where lower scores correspond to poorer quality of life [24].

Furthermore, we considered it appropriate to know the caregiver’s perception of the improvement in patient quality of life as a result of the use of thickeners (previous choking, safety perceived by the caregiver, and quality of life perceived by the caregiver). The validated Zarit questionnaire (ZQ) has demonstrated its efficacy in assessing family claudication in caregivers of dementia patients. In this respect, to assess caregiver burden, we used the short version of the ZQ [25]. This questionnaire consists of 7 items rated as: 1 = never, 2 = almost never, 3 = sometimes, 4 = frequently and 5 = almost always. The cut-off score for defining family claudication was ≥17.

All the described variables, with the exception of the laboratory test data, were collected through interviews lasting approximately 1–2 h via video call to the patient and/or caregiver. The first contact was made by phone, explaining the purpose of the study and its implications, both to the patient with OD and to his or her primary caregiver, requesting an e-mail address where all the information was sent in writing, and signing of the informed consent was requested. After receiving the signed informed consent document, a second call was made to arrange the first video interview, reminding them that they required a measuring tape and scale for body weight for that day. In the first video interview, the MNA and SWAL-QOL were administered. Keeping the video could have favored correct measurement of the anthropometric parameters (the caregiver carried out the different measurements guided by the interviewer—the indications being based on the recommendations of the Spanish Society for the Study of Obesity [26]). In addition, at this time, we confirmed whether the patient had undergone recent laboratory testing and if not, testing was requested, and the patient was instructed to call the nurse of his or her primary care center to make the appointment for collecting the sample at home, after an overnight fast of 10–12 h (the samples were processed in the reference laboratory of the VAUCH). This occasion was also used to arrange a final interview, in this case, addressed to the caregiver, giving him or her the possibility to choose between a phone call or a video call to complete the ZQ. Finally, after obtaining the results, a final telephone contact was made to share the results and offer any relevant dietary advice, as well as prescribe oral nutritional supplementation when considered appropriate.

### 2.4. Statistical Analysis

The data were analyzed using the SPSS version 24.0 statistical package for MS Windows (SPSS, Chicago, IL, USA). Basic descriptive statistics and frequencies were calculated for the different study variables. Nonparametric tests were used to determine potential associations between the study variables (Chi-square test, Kruskal–Wallis test, and Mann–, Whitney U-test), with analysis of correlations to detect relationships or interactions between the different quantitative variables. Statistical significance was considered for *p* < 0.05.

## 3. Results

### 3.1. Demographic and Clinical Characteristics of the Study Population

Thirty-three older patients with OD were recruited (54.5% women), with a mean age of 83.5 ± 7.6 years. The main cause of OD in the study population was neurodegenerative disease (51.5%), followed by cerebrovascular disease (33.3%) and other causes (15.2%). Up to 60% of the patients had suffered respiratory infection before the prescription of the fluid thickener. Additionally, 75.8% of the subjects presented AHT, 54.5% dyslipidemia (DLP), and 51.5% presented DM. Of the 33 subjects, only three did not need the presence of a caregiver. A total of 54.5% of the patients were cared for by an informal caregiver, predominantly female (77.8%) and with a mean age of 66.6 ± 11.1 years (maximum caregiver age: 83 years). Of the informal caregivers, 72.2% acknowledged having claudicated, with an average ZQ score of 22.8 ± 8.0 points.

### 3.2. Adaptation of Solid Texture and Liquid Viscosity

Of the study participants, 21.2% had no teeth, and 26% used teeth (33.3% own dentures and 45.5% dentures). According to diets with modified texture, 72.7% of the patients required a pureed diet, 12.1% an easy-to-chew diet, and 15.2% could eat a regular diet. A total of 57.6% of the patients were totally dependent for eating.

Regarding viscosity to ensure safe swallowing, 97% of the patients required slightly thick viscosity (100 m Pas), and only one patient needed extremely thick viscosity (4500 m Pas).

It should be pointed out that the classification used for the viscosity and texture of foods and beverages is based on the classification established by the International Dysphagia Diet Standardisation Initiative (IDDSI).

### 3.3. Nutritional Status

Regarding the anthropometric parameters, the mean arm circumference was 27.2 ± 6.1 cm, calf circumference 30.3 ± 5.3 cm, body weight 63.9 ± 13.8 kg, and BMI 24.3 ± 6.4 kg/m^2^. In reference to the biochemical parameters, the mean total proteins concentration was 6.5 ± 0.6 g/dL, albumin 3.8 ± 0.5 g/dL and total cholesterol 152.0 ± 36.2 mg/dL (Table 1). Albumin was the only parameter showing a significant trend with age (r: −0.352, *p* = 0.052).

According to the MNA, 60.6% of the patients were at risk of malnutrition, 23.7% were malnourished, and only 12.1% presented a normal nutritional status. Only 24% of the patients were taking oral nutritional supplements. The MNA score was significantly correlated to albumin (r: 0.600, *p* < 0.001) and total proteins (r: 0.435, *p* = 0.015), but not to total cholesterol (r: −0.116, *p* = 0.534) or lymphocytes (r: −0.056, *p* = 0.758). Likewise, total proteins and albumin were correlated (r: 0.706, *p* < 0.001), but neither showed a correlation to total cholesterol (r: 0.156, *p* = 0.402 and r: 0.252, *p* = 0.269, respectively). However, lymphocytes correlated to both total proteins and total cholesterol (r: 0.437, *p* = 0.014 and r: 0.355, *p* = 0.050, respectively) but not to albumin (r: 0.238, *p* = 0.197).

Of the study participants, 30.3% had edemas, 21.2% had a pressure ulcer, and 30.3% had a loss of muscle mass. No significant differences were observed between edema (U: 82.0, *p* = 0.195), pressure ulcer (U: 64.5, *p* = 0.242), or loss of mass muscle (U: 72.5, *p* = 0.095) and malnutrition.

However, 81.8% of the patients considered that they had no malnutrition problems, and only 33.3% considered themselves to be worse than other people of the same age. No positive correlation was observed between nutritional status (MNA score) and patient perception of personal nutritional status (X^2^: 4.009, *p* = 0.405, k = 0.54) (Table 2).

### 3.4. Quality of Life

All caregivers reported perceived improvement in the quality of life of their family member and perceived improvement in safety since the patient took the thickener.

The mean total score of the SWAL-QOL was 75.1 ± 16.4 points.

Significant differences according to the type of diet were found in the mean SWAL-QOL score, with a score of 71.3 ± 15.1 for the pureed diet versus 85.2 ± 16.1 for the minced and moist or regular diet (U: 50.0, *p* = 0.019). Significant differences were also found regarding ONS intake, with a mean SWAL-QOL score of 60.8 ± 13.6 for those supplemented versus 79.7 ± 14.6 for those not supplemented (U: 32.5, *p* = 0.005).

In relation to the mean SWAL-QOL score for comorbidities, a trend towards statistical significance was recorded with respect to patients who presented AHT compared to those who did not (78. 5 ± 14.4 vs. 64.8 ± 18.7, U: 55.0, *p* = 0.059), with no finding differences in DM (75.8 ± 15.7 vs. 74.4 ± 17.5, U: 131.5, *p* = 0.871) or DLP (76.1 ± 17.2 vs. 73.9 ± 15.8, U: 123.5, *p* = 0.677). The mean scores of the SWAL-QOL are reported in Table 3.

In relation to the sex of the patient, a significant difference was observed in the duration of intake dimension, with males obtaining an average score of 4 ± 0.8 points and females 3 ± 1.3 points (U: 78.5, *p* = 0.036). Regarding the sex of the caregiver, significant differences were only obtained in the duration of intake dimension (males 1.5 ± 0.6 versus females 3.7 ± 0.9 (U: 79.5, *p* = 0.009)). The fatigue dimension was significantly different according to the type of caregiver (informal caregiver 3.2 ± 0.9 versus formal caregiver 3.8 ± 1.1 (U: 81.5, *p* = 0.047)).

On grouping according to the type of diet of the patient, relevant differences were found in relation to the frequencies of the symptoms (mashed 3.7 ± 0.8 versus minced and moist diet or regular diet 4.3 ± 0.9 (U: 57.0, *p* = 0.039)) and communication (mashed 3.0 ± 1.5 versus minced and moist diet or regular diet 4.3 ± 0.9 (U: 53.5, *p* = 0.023)).

On classifying according to the presence of pressure ulcers, significant differences were only found with the presence of symptoms (pressure ulcer 3.1 ± 0.9 versus no pressure ulcer 4.1 ± 0.8 (U: 32.5, *p* = 0.010).

When the sample was divided according to ONS intake, significant differences were found among the following dimensions: overload (ONS intake 2.5 ± 1.7 versus no intake 3.7 ± 1.1 (U: 52.0, *p* = 0.037)), appetite (ONS intake 2.6 ± 0.7 versus no intake 4.2 ± 1.2 (U: 31.0, *p* = 0. 002)), frequency of symptoms (ONS intake 3.2 ± 0.9 versus non-intake 4.1 ± 0.8 (U: 43.5, *p* = 0.017)), food selection (ONS intake 3. 5 ± 1.8 versus no intake 4.8 ± 0.5 (U: 61.0, *p* = 0.036)) and mental health (ONS intake 2.4 ± 1.2 versus no intake 3.9 ± 1.3 (U: 42.0, *p* = 0.011)).

The dimensions which yielded significant differences according to the diagnosis of AHT were length of intake (AHT 3.7 ± 1.1 versus no AHT 2.7 ± 1.2 (U: 53. 5, *p* = 0.045)), appetite (AHT 4.2 ± 1.2 versus non AHT 2.8 ± 1.3 (U: 43.5, *p* = 0.013)) and food selection (AHT 4.7 ± 0.9 versus non AHT 3.6 ± 1.3 (U: 39.0, *p* = 0.001)). However, only food selection (DM 4.7 ± 0.9 versus non DM 4.1 ± 1.1 (U: 79.5, *p* = 0.009) yielded significant differences regarding the variable DM.

No dimension reached statistical significance on categorizing the sample according to the diagnosis of DLP. 

In relation to the biochemical parameters, lymphocytes alone were significantly correlated to the dimensions length of intake (r: −0.397, *p* = 0.22) and appetite (r: −0.389, *p* = 0.25).

Patient age showed a significant correlation to food selection (r: 0.520, *p* = 0.002).

There were no statistically significant differences regarding the following variables: dentition, edema, pressure ulcer, loss of muscle mass, nutritional status (MNA score), history of choking, primary diagnosis, level of dependence, or family claudication. Table 4 establishes the correlations between the different dimensions of SWAL-QOL, in which * is identified with a significant correlation coefficient at the level of 0.05; ** at the level of 0.01; and *** at the level of 0.001.

### 3.5. Informal Caregiver Claudication (Zarit Questionnaire)

Significant differences were not obtained for any of the questions of the ZQ regarding sex, ONS intake, dentition status, edema, loss of muscle mass, MNA score, AHT, DM, DLP, history of choking, primary diagnosis, level of dependency, or sex of the caregiver.

For the pressure ulcer groups, a tendency towards statistical significance was found related to the total ZQ score (U: 13.0, *p* = 0.054).

On classifying according to the type of diet, significant differences were recorded with the questions Zarit 2 (pureed 3.7 ± 1.1 versus minced and moist diet or regular diet 2.0 ± 0.0 (U: 4.5, *p* = 0.027)), Zarit 3 (pureed 3.7 ± 1.3 versus minced and moist diet or regular diet 1.7 ± 0.6 (U: 4.0, *p* = 0. 023)), Zarit 6 2 (pureed 3. 8 ± 1.1 versus diet minced and moist or regular diet 2.0 ± 1.0 (U: 5.5, *p* = 0. 038)), Zarit 7 2 (pureed 3.4 ± 1.1 versus diet minced and moist or regular diet 1. 7 ± 0.6 (U: 4.0, *p* = 0. 024)) and Zarit total 2 (pureed 24.7 ± 7.3 versus diet minced and moist or regular diet 13.7 ± 5.1 (U: 5.0, *p* = 0. 038)).

### 3.6. Efficiency of Telematic Consultation (Video Call)

The telematic consultation (video call) carried out by the nutrition unit was effective; none of the patients were admitted to hospital due to respiratory infection or other complications of OD during the second wave of COVID-19.

## 4. Discussion

The increase in life expectancy among the older population has been related to an increase in the prevalence of geriatric syndromes. Currently, OD is recognized by some authors as one of these syndromes [27,28], along with frailty and cognitive impairment [29]. The prevalence of OD in elderly people is high and further increases in the presence of cognitive impairment [4,6,10]).

The prevalence of OD in our population over 65 years of age was 2.14%, and over half of the patients presented severe cognitive impairment (59.1%). The main cause of OD in the study population was neurodegenerative disease (51.5%), followed by cerebrovascular disease (33.3%) and other causes (15.2%). Although with different percentages, these causes are similar to those reported in other studies [30,31].

Dentition is an important factor in the chewing of food and has a decisive influence upon OD. In our sample, 21.2% had no teeth, and 78.8% wore dentures, which may be a factor in favor of OD. In the study by Inui et al. [32], the number of teeth in the men in their sample was significantly associated with OD (odds ratio (OR) = 0.946, 95% confidence interval (CI), *p* = 0.038)). Similarly, Okabe et al. [33] found the risk of OD to decrease in those individuals with the greatest number of functional teeth (OR = 0.92, 95% confidence interval (CI) 0.87–0.98).

In the current world health situation, where hospitals are saturated as a result of priority attention to patients with COVID-19, especially in services that focus on respiratory disease [34], it has become a priority to avoid the readmission of elderly patients with OD presenting one of its potential complications: respiratory infection. It should be noted that 60% of the subjects in our study had a history of at least one admission due to respiratory infection before the prescription of the thickener. Martin et al. [35] published a clinical trial in which the intervention targeted to the control group included, among other measures, the use of thickeners and the modification of food texture. The incidence of respiratory infections with readmission to hospital was 12.5% in the intervention group versus 74.68% in the control group (*p* = 0.002). Taking into account the physiological characteristics of the sample, the use of thickeners in the long term was shown to be the safest option to avoid respiratory complications, with the capacity of the deglutition muscles being difficult to recover in elderly patients with neurodegenerative diseases. Similarly, in our study, after the prescription of the thickener and the change in viscosity to ensure safe swallowing (97% needed slightly thick viscosity and only one patient required extremely thick viscosity), all of the subjects reported an increase in their sense of eating safety. Similarly, telematic visits via video call were an essential aspect in increasing the feeling of improvement in the state of health on the part of the patients. In spite of this, 60.6% presented a risk of malnutrition. Caregivers reported a decrease in signs by decreasing cough, number of choking episodes, fear of food intake..., as shown in the SWAL-QO questionnaire, as well as a decrease in hospital admissions for consequent respiratory infections. In addition, caregivers perceived more confidence in themselves to feed their family members. This observation is consistent with the data published by other authors [36,37,38].

Another of the most frequent complications in older patients with OD is malnutrition [39]. The data obtained support the results of previous research [40,41,42], with only 12.1% of the elderly with OD having optimal nutritional status according to the MNA. In our study, a significant correlation was obtained between the MNA score and the levels of serum albumin (r: 0.600, *p* < 0.001) and total proteins (r: 0.435, *p* = 0.015), but not between the MNA score and total cholesterol (r: −0.116, *p* = 0.534) or lymphocytes (r: −0.056, *p* = 0.758). This significant correlation to albumin and total proteins was also recorded in the study by Zhang et al. [43], where subjects at high risk of malnutrition yielded significantly lower figures referred to BMI, serum albumin, hemoglobin, total cholesterol, prealbumin, and total proteins compared with individuals without risk of malnutrition. Miao et al. [44] likewise confirmed a significant decrease in hemoglobin, albumin, prealbumin, and lymphocytes in subjects with malnutrition.

Albumin, the biochemical, nutritional reference parameter, was the only parameter showing a significant trend with age (r = −0.352, *p* = 0.052). This observation coincides with the results published by Wu et al. [45], who found the albumin levels to tend to decrease with advancing age in older people. In contrast, however, different authors have reported that blood albumin concentration is not directly related to age but to other aspects, such as the diet or socioeconomic level of the population [46,47]. Byun et al. [48] found serum albumin < 3.5 g/dL to be an independent risk factor for OD.

Nutritional status, as reflected by the MNA, was not positively correlated to age (r: −0.227, *p* = 0.204). Likewise, there was no positive correlation between nutritional status as measured by the MNA and patient-perceived nutritional status (81.8% of the subjects did not consider themselves to have malnutrition problems). In the study by Gyan et al. [49], patients and families underestimated their own and their family member’s malnutrition, respectively (*p* < 0.001). At this point, it is important to reflect on current community health care, where to avoid the movement of patients at risk to health centers, a telephone service without image (call) has been set up. However, the results of this research have shown that patients may overestimate their state of health, suggesting the need for continuous re-evaluation by the specialized units supported by image calls and measurement of objective parameters.

Malnutrition or the risk of malnutrition influences the quality of life of older people regardless of whether they have OD [50] or not [51] and affects their ability to perform basic activities of daily living (BADL) with autonomy. In our study, 57.6% of the sample needed help to feed themselves, while 72.7% were dependent in all or some areas of BADL. This dependence, together with the advanced age of the subjects, resulted in 33.3% of them considering their state of health to be worse than that of the rest of people of their age. Different factors can intervene in the self-perception of health, though a key element, according to several studies [52,53], is represented by the actual health problems or health conditions suffered by the person.

OD also inevitably influences the quality of life of patients. Kim et al. [54] studied stroke patients with OD and recorded a significant negative correlation between the quality of life (SWAL-QOL) and the degree of OD (r = −0.468, *p* = 0.012).

In our study, patients on an easy-to-chew or unmodified diet had a better quality of life than those on a pureed or slightly thick diet. In the clinical trial carried out by Reyes-Torres et al. [55], statistically significant improvements were recorded in biochemical, body, and muscle function parameters after modifying the texture and viscosity (slightly thick and extremely thick) of the diet of patients with OD. In contrast, the reviews by O’Keeffe [56] and Beck et al. [57] showed that there is not enough scientific evidence to confirm that changes in diet texture and viscosity of people with OD reduce the risk of food aspiration or pneumonia, or their quality of life.

The use of nutritional supplements can have beneficial effects on health. In the clinical trial carried out by Martin et al. [35], caloric and protein supplementation, in addition to the use of thickeners, texture modification, and improved oral hygiene, yielded improvements in nutritional status, functionality, and reduction in respiratory infections, hospital admissions and mortality in older people with OD. In our study, self-perceived quality of life (SWAL-QOL) among the patients who did not take supplements was higher than in those who did. Patients who do not take supplements perceive less emotional overload, a greater desire to eat, less frequent occurrence of OD symptoms, less difficulty in food choice, and fewer psychological health problems compared to patients who do take supplements.

The evidence on SWAL-QOL is limited. In this regard, no previous studies have been found that could support the relationships observed between the sex of the caregiver and the duration of intake, or the perception of fatigue in patients with informal caregivers, among others.

It should be noted that the fact that most of the subjects were dependent for activities of daily living conditioned the presence of the caregiver. In our study, informal caregivers predominated over formal caregivers. In the case of the informal caregivers, the family dependency situation led to claudication of the caregivers—most of which were women (77.8%)—which in turn influenced their quality of life and health. On further adding the situation of the current COVID-19 pandemic, increased overload could result, as measured by the ZQ. The main role of women as informal caregivers coincides with the observations of Xiong et al. [58] and Abdel-Malek et al. [59]. Due to the current pandemic situation and the overloading of primary-care level facilities, follow-up of high-risk dependent patients has been reduced, as has support for the caregiver. In addition, the lack of training of primary care professionals in the management of OD makes it even more difficult to manage the problems that it can cause and to stop it early to avoid infections and admissions, among others. For this reason, telematic consultation via video call should be established to follow up chronic high-risk patients, as is the case in our sample, both in primary care and in any hospital specialty. On the other hand, communication between primary-care level (care providers without specific training in OD) and hospital professionals becomes relevant to be able to refer patients who need it in time. In this way, it would be possible to detect early the need for an assessment by the specialist, such as an increased incidence of safety signs in swallowing, such as coughing, choking, wet voice, etc., and thus be able to refer these patients to the nutrition consultation to re-evaluate the viscosity and texture they need.

We observed a trend towards statistical significance in the association between patients with pressure ulcers and informal caregiver self-reported fatigue. Pressure ulcers are often long-lasting and heal poorly in older people, and this fact, among others, could explain the physical and psychological fatigue of the caregiver [60]—especially if no external formal or informal help is available.

We did not record statistical significance for the relationship between malnutrition and pressure ulcers or loss of muscle mass, in contrast to the observations of other studies [61,62].

The type of diet with pureed texture versus the diet with minced and moist or regular diet yielded significant differences with ZQ questions 2, 3, 6, 7, and the total score of the questionnaire. We have found no references in the scientific literature regarding these aspects, and future studies are therefore needed to address these issues.

At present, due to the COVID-19 pandemic, effective telematic health care is required in specialized hospitalization units to avoid face-to-face care of high-risk patients and their readmission due to complications. Thus, this study carried out by the VAUCH Nutrition Unit has demonstrated the need and viability to maintain a re-evaluation of the nutritional status in OD patients with a telematics system with image (video call) that allows the measurement of subjective and objective parameters, since the limitation of telematic care by voice could limit the detection of risk states (60.6% of patients presented a risk of malnutrition according to MNA) versus the verbalization of considered that they had no malnutrition problems (81.8%) and a good quality of life (SWAL-QOL was 75.1 ± 16.4 points); supported all caregivers reported perceived improvement in the quality of life of their family member and perceived improvement in safety since the patient took the thickener... On the other hand, the results obtained on ZQ remind us of the importance of caring for the caregiver (of the informal caregivers, 72.2% acknowledged having claudicated, with an average ZQ score of 22.8 ± 8.0 points).

Among the limitations of the study, mention must be made of the small sample size. However, previous research has evidenced the difficulty of recruiting older patients with OD without cognitive impairment or with only mild impairment allowing the assessment of quality of life in relation to OD [63]. Future research should corroborate the indications provided by this study, adopting a multicenter design to recruit a larger number of patients.

## Figures and Tables

**Table 1:** Biochemical parameters.

Biochemical Parameters	*N*	Minimum	Maximum	*M* ± *SD*
Glucose (mg/dL)	33	62.00	225.00	119.4 ± 41.8
Urea (mg/dL)	33	17.00	82.00	44.5 ± 14.8
Creatinine (mg/dL)	33	0.51	62.00	2.9 ± 10.6
Uric acid (mg/dL)	32	2.00	10.50	5.3 ± 1.9
Total proteins (g/dL)	31	4.70	7.80	6.5 ± 0.7
Albumin (g/dL)	31	2.40	4.70	3.8 ± 0.5
Calcium (mg/dL)	30	7.20	10.20	9.1 ± 0.6
Phosphorus (mg/dL)	22	1.80	4.70	3.3 ± 0.8
Bilirubin (mg/dL)	32	0.14	.86	0.4 ± 0.2
Sodium (mEq/L)	33	133.00	146.00	141 ± 3.0
Potassium (mEq/L)	33	3.60	5.60	4.5 ± 0.5
Chloride (mEq/L)	33	93.00	109.00	101.2 ± 3.8
Iron (µg/dL)	22	20.00	140.00	66.8 ± 31.3
Ferritin (ng/mL)	30	14.00	549.00	136.5 ± 133.3
Transferrin (mg/dL)	17	123.00	315.00	214.7 ± 54.0
Transferrin saturation (%)	16	7.30	45.60	19.4 ± 10.7
Folate (ng/mL)	18	3.20	23.00	9.9 ± 5.9
Vitamin B12 (pg/mL)	23	175.00	760.00	514.4 ± 188.2
Triglycerides (mg/dL)	31	43.00	526.00	141.0 ± 99.1
Cholesterol (mg/dL)	31	91.00	234.00	152.0 ± 36.2
HDL-cholesterol (mg/dL)	25	25.00	81.00	47.6 ± 16.3
LDL-cholesterol (mg/dL)	25	7.00	143.00	73.6 ± 31.7
GGT (U/L)	30	5.00	158.00	31.4 ± 35.5
Alkaline phosphatase (U/L)	32	45.00	190.00	86.2 ± 32.5
Creatine kinase (U/L)	25	14.00	292.00	65.5 ± 57.0
GOT (AST) (U/L)	33	10.00	33.00	17.7 ± 5.0
GPT (ALT) (U/L)	32	5.00	39.00	13.9 ± 8.0
C-reactive protein (mg/dL)	28	0.21	11.81	2.6 ± 3.3
MDRD (mL/min/1.73 m^2^)	26	40.82	182.52	69.7 ± 32.7
CKD-EPI equation (mL/min/1.73 m^2^)	30	34.74	99.34	62.9 ± 19.8
TSH (µU/mL)	29	0.93	6.80	2.24 ± 1.2
Glycosylated hemoglobin (%)	23	5.30	9.50	6.7 ± 1.2

**Table 2:** Cross-tabulation of MNA O and MNA*MNA_DIAGNOSIS.

			MNA_DX			Total
			NORMAL	RISK	MALNUT	
**Subjective** **Malnutrition**	***NORMAL***	Count	4	17	6	27
		% within subjective malnutrition	14.8%	63.0%	22.2%	100.0%
		% within MNA_DX	100.0%	85.0%	66.7%	81.8%
		% of total	12.1%	51.5%	18.2%	81.8%
	**RISK**	Count	0	3	2	5
		% within subjective malnutrition	0.0%	60.0%	40.0%	100.0%
		% within MNA_DX	0.0%	15.0%	22.2%	15.2%
		% of total	0.0%	9.1%	6.1%	15.2%
	**MALNUT**	Count	0	0	1	1
		% within subjective malnutrition	0.0%	0.0%	100.0%	100.0%
		% within MNA_DX	0.0%	0.0%	11.1%	3.0%
		% of total	0.0%	0.0%	3.0%	3.0%
**Total**		Count	4	20	9	33
		% within subjective malnutrition	12.1%	60.6%	27.3%	100.0%
		% within MNA_DX	100.0%	100.0%	100.0%	100.0%
		% of total	12.1%	60.6%	27.3%	100.0%

**Table 3:** The Swallowing Quality of Life (SWAL-QOL) results.

DOMAINS	M ± SD
**OVERLOAD**	3.4 ± 1.4
It is very difficult for me to bear my swallowing problem	3.4 ± 1.4
My swallowing problem is a major concern in my life	3.5 ± 1.4
**DURATION OF INTAKE**	3.4 ± 1.2
I take longer to eat than others	3.4 ± 1.3
It takes me a long time to finish a meal	3.5 ± 1.3
**APPETITE**	3.8 ± 1.3
Most days, I don’t care whether I eat or not	3.5 ± 1.3
I no longer enjoy eating	3.8 ± 1.4
I am hardly ever hungry anymore	3.8 ± 1.4
**FREQUENCY OF SYMPTOMS**	3.9 ± 0.9
I cough	3.6 ± 1.4
Choking on solid food	3.8 ± 1.4
Choking on liquid food	3.7 ± 1.3
Saliva or thick phlegm	3.7 ± 1.4
I choke when I eat	3.8 ± 1.3
Excess saliva or phlegm	3.4 ± 1.5
Having to clear my throat (throat clearing)	3.6 ± 1.5
Drooling	4.1 ± 1.4
Problems when chewing	2.6 ± 1.8
Food residues in the throat	4.2 ± 1.1
Food residues in the mouth	4.4 ± 1.1
Solids and liquids coming out of the mouth	4.4 ± 1.1
Solids and liquids coming out of the nose	4.4 ± 1.1
Coughing up food or liquid	4.5 ± 0.9
**SELECTION OF FOOD**	4.5 ± 1.1
I have a problem thinking about what I can eat	4.5 ± 1.1
It is difficult to find foods that I like and can eat	4.5 ± 1.1
**COMMUNICATION**	3.4 ± 1.5
Others find it difficult to understand me when I speak	3.5 ± 1.5
I find it difficult to speak clearly	3.4 ± 1.5
**FEARS**	4.0 ± 1.4
I am afraid of choking when I eat	3.9 ± 1.3
I am worried about having pneumonia	4.0 ± 1.3
I am afraid to choke when I drink	4.0 ± 1.3
I never know when I am going to choke	4.0 ± 1.2
**MENTAL HEALTH**	3.5 ± 1.4
My swallowing problem depresses me	3.5 ± 1.4
My swallowing problem irritates me	3.5 ± 1.4
It bothers me to have to take so many precautions when eating or drinking	3.5 ± 1.4
My swallowing problem frustrates me	3.5 ± 1.4
I am discouraged because of my swallowing problem	3.5 ± 1.4
**SOCIAL**	3.6 ± 1.3
I do not go out to eat because of my swallowing	3.6 ± 1.3
My swallowing problem makes my social life difficult	3.6 ± 1.3
My usual activities have changed due to my swallowing problem	3.7 ± 1.3
I do not enjoy social gatherings because of my swallowing problem	3.7 ± 1.3
My role with my family and friends has changed due to my swallowing problem	3.7 ± 1.4
**FATIGUE**	3.5 ± 1.0
I feel weak	3.3 ± 1.1
I feel tired	3.6 ± 1.1
I feel exhausted	3.6 ± 1.1
**SLEEP**	3.9 ± 1.1
I have trouble sleeping	3.9 ± 1.1
I have trouble keeping asleep	3.9 ± 1.1

**Table 4:** Correlations between the different dimensions of the SWAL-QOL.

		D1	D2	D3	D4	D5	D6	D7	D8	D9	D10	D11
**D1**	Pearson correlation	1	0.286	0.444 **	0.580 **	0.251	0.401 *	0.398 *	0.701 **	0.426 *	0.381 *	−0.116
	Sig. (2-tailed)		0.106	0.010	0.000	0.159	0.021	0.022	0.000	0.013	0.029	0.520
	N	33	33	33	33	33	33	33	33	33	33	33
**D2**	Pearson correlation	0.286	1	0.723 **	0.274	0.292	0.371 *	0.344 *	0.142	0.311	0.317	0.147
	Sig. (2-tailed)	0.106		0.000	0.122	0.099	0.034	0.050	0.430	0.078	0.072	0.415
	N	33	33	33	33	33	33	33	33	33	33	33
**D3**	Pearson correlation	0.444 **	0.723 **	1	0.470 **	0.493 **	0.221	0.428 *	0.357 *	0.383 *	0.381 *	−0.002
	Sig. (2-tailed)	0.010	0.000		0.006	0.004	0.217	0.013	0.041	0.028	0.029	0.992
	N	33	33	33	33	33	33	33	33	33	33	33
**D4**	Pearson correlation	0.580 **	0.274	0.470 **	1	0.364 *	0.342	0.763 **	0.714 **	0.558 **	0.466 **	−0.026
	Sig. (2-tailed)	0.000	0.122	0.006		0.037	0.051	0.000	0.000	0.001	0.006	0.888
	N	33	33	33	33	33	33	33	33	33	33	33
**D5**	Pearson correlation	0.251	0.292	0.493 **	0.364 *	1	−0.199	0.262	0.473 **	0.217	0.071	−0.002
	Sig. (2-tailed)	0.159	0.099	0.004	0.037		0.267	0.141	0.005	0.224	0.695	0.990
	N	33	33	33	33	33	33	33	33	33	33	33
**D6**	Pearson correlation	0.401 *	0.371 *	0.221	0.342	−0.199	1	0.448 **	0.345 *	0.496 **	0.328	0.345 *
	Sig. (2-tailed)	0.021	0.034	0.217	0.051	0.267		0.009	0.049	0.003	0.062	0.049
	N	33	33	33	33	33	33	33	33	33	33	33
**D7**	Pearson correlation	0.398 *	0.344 *	0.428 *	0.763 **	0.262	0.448 **	1	0.648 **	0.687 **	0.135	−0.014
	Sig. (2-tailed)	0.022	0.050	0.013	0.000	0.141	0.009		0.000	0.000	0.453	0.938
	N	33	33	33	33	33	33	33	33	33	33	33
**D8**	Pearson correlation	0.701 **	0.142	0.357 *	0.714 **	0.473 **	0.345 *	0.648 **	1	0.731 **	0.301	0.061
	Sig. (2-tailed)	0.000	0.430	0.041	0.000	0.005	0.049	0.000		0.000	0.089	0.734
	N	33	33	33	33	33	33	33	33	33	33	33
**D9**	Pearson correlation	0.426 *	0.311	0.383 *	0.558 **	0.217	0.496 **	0.687 **	0.731 **	1	0.363 *	0.152
	Sig. (2-tailed)	0.013	0.078	0.028	0.001	0.224	0.003	0.000	0.000		0.038	0.399
	N	33	33	33	33	33	33	33	33	33	33	33
**D10**	Pearson correlation	0.381 *	0.317	0.381 *	0.466 **	0.071	0.328	0.135	0.301	0.363 *	1	0.144
	Sig. (2-tailed)	0.029	0.072	0.029	0.006	0.695	0.062	0.453	0.089	0.038		0.425
	N	33	33	33	33	33	33	33	33	33	33	33
**D11**	Pearson correlation	−0.116	0.147	−0.002	−0.026	−0.002	0.345*	−0.014	0.061	0.152	0.144	1
	Sig. (2-tailed)	0.520	0.415	0.992	0.888	0.990	0.049	0.938	0.734	0.399	0.425	
	N	33	33	33	33	33	33	33	33	33	33	33

## Data Availability

Data is contained within the article or supplementary material.
